# Collaborative Work with Highly Automated Marine Navigation Systems

**DOI:** 10.1007/s10606-022-09450-7

**Published:** 2022-10-08

**Authors:** Erik Veitch, Henrikke Dybvik, Martin Steinert, Ole Andreas Alsos

**Affiliations:** 1 Department of Design, Faculty of Architecture and Design, Norwegian University of Science and Technology (NTNU), Produktdesign, 341, Gløshaugen, Kolbjørn Hejes Vei 2 B, 7491 Trondheim, Norway; 2 Department of Mechanical and Industrial Engineering, Faculty of Engineering, Norwegian University of Science and Technology (NTNU), Verkstedteknisk, P317, Gløshaugen, Richard Birkelandsvei 2B, 7034 Trondheim, Norway

**Keywords:** Collaborative work, Interaction design, Navigation, Human–computer interaction, Autonomous ships, Artificial intelligence, Control rooms

## Abstract

In navigation applications, Artificial Intelligence (AI) can improve efficiency and decision making. It is not clear, however, how designers should account for human cooperation when integrating AI systems in navigation work. In a novel empirical study, we examine the transition in the maritime domain towards higher levels of machine autonomy. Our method involved interviewing technology designers (n = 9) and navigators aboard two partially automated ferries (n = 5), as well as collecting field observations aboard one of the ferries. The results indicated a discrepancy between how designers construed human-AI collaboration compared to navigators’ own accounts in the field. Navigators reflected upon their role as one of ‘backup,’ defined by ad-hoc control takeovers from the automation. Designers positioned navigators ‘in the loop’ of a larger control system but discounted the role of in-situ skills and heuristic decision making in all but the most controlled takeover actions. The discrepancy shed light on how integration of AI systems may be better aligned to human cooperation in navigation. This included designing AI systems that render computational activities more visible and that incorporate social cues that articulate human work in its natural setting. Positioned within the field of AI alignment research, the main contribution is a formulation of human-AI interaction design insights for future navigation and control room work.

## Introduction

High levels of machine autonomy and Artificial Intelligence (AI) have the potential to improve work efficiency and improve human decision making. McCarthy (2007) defined AI as ‘the science and engineering of making intelligent machines,’ and intelligence as ‘the computational part of the ability to achieve goals in the world.’ Since the field’s inception in the 1950s, one of the frontiers of AI research has been navigation. Navigation – the process of moving a vehicle from one place to another – exemplifies the primary goal of computational intelligence: the capacity to execute planned action, as if by its own agency. In this study, we examine a transition currently underway in maritime navigation – a transition characterized by increasingly high levels of machine autonomy and incorporation of AI tools designed to collaborate with skilled navigators. Given the breakthroughs in AI technology in the past decade, we explore the extent to which a new human–machine interface is at hand and the extent to which systems design must realign to demands underlying a new order of work.

Driven by advances in computational power and the availability of hardware, examples of high levels of autonomy and AI in maritime applications are becoming more commonplace. Autonomous Surface Vehicles (ASVs) are plying the oceans for scientific data (e.g., Dallolio et al., 2019; Dunbabin et al., 2009; Kimball et al., 2014), autonomous passenger ferries are offering new alternatives to urban mobility (e.g., Reddy et al., 2019; Wang et al., 2019; MiT, 2020; Reddy et al., 2019; Wang et al., 2019), and Maritime Autonomous Surface Ships (MASSs) are introducing new ways to transport payload more efficiently across integrated ports (e.g., Burmeister et al., 2014; Peeters et al., 2020) In this study, we look at the case of partially automated Roll-On/Roll-On (Ro-Ro) ferries operating in Norway, where navigators complete crossings and dockings at the press of a few buttons (e.g., Kongsberg, 2020; Rolls-Royce, 2018). Looking ahead, we can expect implementation of machine learning tools designed to aid navigators make decisions (e.g., Martinsen & Lekkas, 2018; Gjærum et al., 2021; Wu et al., 2021), and computer vision to identify targets and automatically avoid collisions (e.g., Brekke et al., 2019; Helgesen et al., 2022).

High levels of autonomy and AI in sociotechnical applications like navigation rely upon collaboration with skilled human operators. ASVs need remote supervision (Utne et al., 2020), urban autonomous passenger ferries need human safety hosts, (Goerlandt and Pulsifer, 2022), and MASSs need supervision and remote control (Veitch and Alsos, 2022). Aboard the partially automated ferries we study in this article, operations depend upon the presence of a navigator who remains responsible for the vessels and its passengers and stands ready to take over control from the automated system. Despite more advanced systems that automate human manual control tasks and support decision making, the transition underway is not one of less human involvement, as one might expect, but of more collaboration between machines and humans. For designers, such systems present significant challenges. Recent accidents in aviation and car automation serve as dramatic examples of how the transition to human–machine collaboration can lead to accidents. In the years 2018 and 2019, two Boeing 737 MAX crashes revealed that the flight crew fatally lost control when counteracting a non-existent stall. A faulty airflow sensor feeding inputs to the Maneuvering Characteristics Augmentation System (MCAS) was to blame: an automated pitch controller that the flight crew did not know how to override due to its hasty implementation (Nicas et al., 2019). In another instance, a fatal Tesla ‘Autopilot’ crash was found to be caused by ‘system limitations’ combined with ‘ineffective monitoring of driver engagement, which facilitated the driver’s complacency and inattentiveness’ (National Transportation Safety Board, 2020, p. 58). As expressed by a leading autonomous car company in their safety report: ‘While the benefits of automation are obvious, it can actually become a problem if people get tired or bored from having too little to do’ (Waymo, 2020, p. 37). Whether it is an airplane, car, or even a ship, those individuals in control are increasingly finding themselves in a supervisory role, a role that Brian Christian has provocatively called the ‘sorcerer’s apprentice.’ ‘We conjure a force, autonomous but totally compliant, give it a set of instructions, and scramble like mad when we realize our instructions are imprecise or incomplete’ (Christian, 2020, p. 31). In sociotechnical systems like that exemplified by a ship, where control is not executed by a single person but a whole team acting as one (Hutchins, 1995), this role, defined by the crossover between human and machine control, presents new challenges when considering work as fundamentally social action.

The premise for our study is that increased collaboration with computationally intelligent machines places new demands on its human counterparts, and that these demands can be discovered through observation and data collections efforts. Framing the current period of transition in maritime navigation as an opportunity to study these new demands, our aim is to incorporate perspectives of navigators experiencing this transition into further design iterations. Motivated by the potential of machine autonomy to enhance work efficiency and improve decision making, we seek to contribute to system design featuring a more seamless interface for coordinating action.

## Related literature

Drawing on computer science, engineering, design, human–computer interaction, and sociology, we explore how current knowledge gaps and issues are compelling a new research direction positioned at these disciplines’ crossroads. The background literature we present here sets the stage for our study, deepening and expanding the discussion about how technology designers are shaping human-AI collaborative work.

### Levels of autonomy and artificial intelligence

AI has no formal definition. Far from presenting a problem for the field’s practitioners, though, this lack of definition has, in the eyes of its leading experts, been precisely what has driven the field forward (Stone et al., 2016). The sociologist Levi Strauss used the term ‘floating signifier’ to describe phenomena like AI which, in evading definition, strengthen its suggestive power (Lechte, 1994, p. 26). The consequence of such a suggestive power, however, is captured in the so-called ‘AI effect,’ which describes the tendency for any new technology produced by the field, once accepted, to cast off its claims to AI. AI, in this sense, is precisely what is under development. In the development of autonomous vehicles, which represents the field’s idyllic mission of imbuing agency in a computational object, traces of the AI effect can be detected in the taxonomies commonly adopted to establish ‘how autonomous’ a vehicle is. These ‘Levels of Autonomy’ (LoA) taxonomies are not binary (autonomous or not) as one might expect. Rather, LoAs are more like standardized yardsticks for the extent to which a vehicle’s agency is independent of the human driver’s. These taxonomies have their origin in road transportation (SAE International, 2017) and have more recently been developed for maritime transportation (IMO, 2018; Rødseth, 2017). While LoA taxonomies vary, their basic structures remain the same, laying out an integer scale starting at zero or one, which represents full human control, and extending incrementally to some number that represents full machine control. For the vast majority of technology developers, this top number, like the field that proposed it, is a floating signifier. Only the intermediate numbers, which presume a collaborative approach to the myriad actions involved in driving a vehicle, are considered feasible.

Despite the apparently intractable goals underlying machine autonomy, the field of AI has been remarkably productive in producing technologies and techniques enabling intermediate LoAs. The theoretical underpinnings of modern computational machine learning techniques like Deep Neural Network (DNNs) have been around for decades, but only in the past decade has computational power enabled their widespread use. Advancements in machine learning techniques, too, have rapidly advanced the field, including in areas like natural language processing, image and video classification and generation, planning, decision making, and integration of vision and robotics. In the face of such advancements, however, a major new challenge has arisen. As expressed in Stanford University’s ‘*AI100 Report*,’ the field’s most influential experts recognized that, ‘Perhaps the most inspiring challenge is to build machines that can cooperate and collaborate seamlessly with humans’ (Littman et al., 2021, p. 19). In response to this challenge, an active research community has sprung up. These researchers are dedicated to ‘AI alignment,’ and include not just computer engineers and designers, but also anthropologists and sociologists, safety specialists and organizational scientists. In the context of sociotechnical systems, like that exemplified by our focus on the transition in maritime navigation, there is a growing need for such multidisciplinary efforts to understand the implications of high levels of autonomy and AI in safety–critical work. We position our work within the efforts of AI alignment research, interpreting the transition underway as one necessitating a realignment of design practices with the social actions coordinating human work.

### Centres of coordination

The supervisory role taking shape in the wake of higher levels of autonomy has generated interest in centres of coordination for autonomous vehicles. For maritime navigation, this is exemplified by the concept of land-based supervisory control of highly automated ships, variously referred to in the literature as ‘shore control centre,’ ‘remote control centre,’ or ‘remote operating centre.’ These terms, which have surfaced in the past decade, capture a renewed interest in control rooms. Control rooms were a topic of academic interest in human factors and cognitive engineering in the 1970s and 80s especially in the context of complex, sociotechnical systems like nuclear power plants (Rasmussen, 1986; Vicente, 1999). In the 1990s, control rooms were of academic interest in the field of Computer Supported Collaborative Work. Researchers in CSCW studied the sociality of computer use in natural settings like line control rooms (Heath & Luff, 1992), airline scheduling (Goodwin & Goodwin, 1996), and emergency dispatch (Whalen, 1995). Today, in the wake of technological developments enabling higher levels of autonomy, the spotlight is once again directed towards the control room, the stage upon which supervisory control and time-critical action is orchestrated, enabling the coordination of highly automated vehicles across distributed locations. In this context, we revisit Lucy Suchman’s definition of ‘centre of coordination’:

‘Centres of coordination are characterized in terms of participants’ ongoing orientation to problems of space and time, involving the deployment of people and equipment across distances, according to a canonical timetable or the emergent requirements of rapid response to a time-critical situation.’ (Suchman, 1997, p. 42)

For autonomous ships, the ‘shore control centre’ as a centre of coordination presents significant challenges to designers. The International Maritime Organization (IMO), the inter-governmental agency for standardisation of safety at sea, outlined such outstanding challenges in their ‘Regulatory scoping exercise for the use of maritime autonomous surface ships (MASS)’ (IMO, 2021). In their report, the highest priority issues concerned the role of the navigator working in a location separate from the ship environment. While navigators’ responsibility for the safety of the ship remained unchanged, the environment in which they work was substituted by an information-rich landscape necessitating new skills and competencies (IMO, 2021, p. 8). In revisiting centres of coordination, we explore what concepts and theories that emerged from seminal control room studies remain relevant today, and what gaps emerge in the light of new technological and organizational developments.

### Design for AI collaborative systems

The need for improved human–machine collaboration predicated by recent technological development has led to new frameworks adopting human-centred design principles to AI systems. Shneiderman (2020), for example, proposed a design framework for ‘human-centred AI’ based on the principles of safe, reliable, and trustworthy system interactions. The field of Human–Computer Interaction (HCI) has put forward practical guidelines for designers adapting to such frameworks (e.g., Amershi et al., 2019; Mahadevan et al., 2018). The rapidly growing field of explainable AI (XAI), too, focuses on the interaction between humans and machines, aiming to establish human-based values of interpretability and understandability at the core of ‘black box’ machine learning techniques (Voosen, 2017). Expanding the audience of XAI towards users, organisations, and even non-governmental agencies, Arrieta Barredo et al. (2020) envision a ‘Responsible AI’ initiative, which embraces values of fairness and accountability along with the mandate of model explainability at the core of XAI. The multidisciplinary field of ‘machine behaviour’ has also emerged recently, which sets out as its mission the empirical treatment of the ways in which human social interactions are modified by the introduction of intelligent machines (Rahwan et al., 2019). The field of CSCW, with its interest in computationally infused environments and enacted elements of work, also stands to offer distinct contributions to this discussion. Ethnomethodological works on social interactions during navigation and control of ships (Hutchins, 1995) and airplanes (Nevile, 2001) lay the theoretical groundwork for such contributions, while more recent discussions exploring ‘ethical AI issues’ (Fleischmann et al., 2019) and ‘challenges in human-AI collaboration’ (Park et al., 2019) pave the way for current research directions. The aim of our study continues in this vein, motivated by lack of knowledge about how the transition to higher levels of autonomy affects the social underpinnings enacting work in its natural setting.

### Ironies of automation

Bainbridge (1983), writing in her seminal paper ‘Ironies of Automation,’ described the paradoxical decrease in human abilities resulting from machines designed to improve that very ability. Among human factors specialists the effect is well-known, but despite its articulation three decades ago, its consequences persist in modern system design. For example, skill degradation associated with automation emerged as a key factor in the high-profile crash of flight Air France flight 447 in 2009, which fatally stalled over the Atlantic Ocean after the automatic flight system handed control to the flight crew shortly after detecting faulty readings from an airspeed sensor. As the accident report stated, one of the contributing causes of the stall and resulting crash was ‘The absence of any training, at high altitude, in manual aeroplane handling’ (Bureau d’Enquêtes et d’Analyses pour la sécurité de l’aviation civile, 2012, p. 201). Consequently, guidelines now recommend pilots practice manual flying regularly, highlighting that ‘continuous use of automation does not strengthen pilots’ knowledge and skills in manual flight operation and in fact could lead to degradation of the pilots’ ability to quickly recover the aircraft from an undesired state’ (IATA, 2020, p. 5). The consequences of skill degradation are exacerbated in systems with high LoA that require timely and decisive preventative action from a skilled operator. Taking irony to be a poor premise for design, we consider instead how design activities better aligned to the needs of collaboration can avoid the pitfalls associated with automation-induced skill degradation.

## Methodology

Our methodology consisted of field study observations and semi-structured interviews. The research design was motivated by the practical need to inform design efforts implementing high levels of machine autonomy and AI techniques in maritime navigation applications. The aim was to describe the extent to which design practices currently shaping a transition in the maritime domain are aligned with the realities of skilled, safety–critical work in the field.

### Data collection

The empirical data consisted of semi-structured interviews with individuals in the design and research communities (n = 9) and navigators working aboard two partially automated passenger ferries (n = 5). To provide context about the natural setting in which the navigation work takes place, we also report on field observations conducted at the site of one of the ferries featured in the interviews. All data were collected in Norway.

Selection of interview participants was guided by theoretical sampling commonly employed in Grounded Theory Methods (Corbin and Strauss, 2015; Glaser and Strauss, 1999). This allowed us to follow up on themes of interest and target subsequent participants as new open-ended questions presented themselves. After completing nine interviews with technology designers and researchers in autumn 2019, it became clear that the perspective of navigators in the field would be of interest. Turning to this gap, a field study was conducted aboard the navigation deck of a ferry outfitted with state-of-the-art automated navigation technology. Field notes and images were collected by the first author, and once again new questions were posed. Interviews were subsequently held in summer 2021 with three of the navigators aboard this ferry, followed by two more aboard a similar ferry.

Two researchers were present for all interviews, with one leading the conversation while the other transcribed, verbatim. The first author was present for all interviews, which were conducted either in-person or via video call and ranging in length from 45 to 60 minutes. Participants consented to data collection before and after the interviews, and all interviews that were held in Norwegian were translated into English.

### Interview respondents

**Figure 1:**
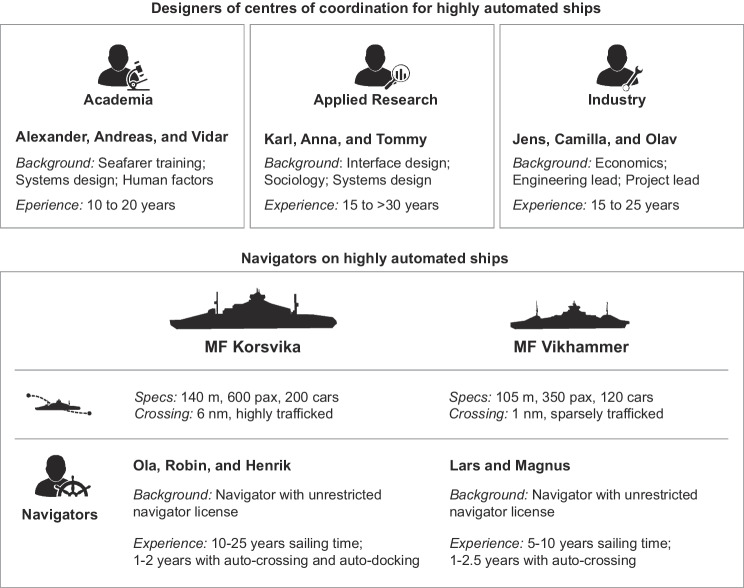
Description of interview informants

A total of fourteen informants were interviewed, each of whom we refer to in this study with a pseudonym (Figure 1). The group whom we refer to as ‘Designers’ originated from academia, applied research, and industry. This group consisted of individuals with expert domain knowledge about the development of centres of coordination for highly automated ships. Their unique contribution was insights about activities shaping the transition towards higher levels of autonomy in marine navigation work. This group captured a wide breadth of perspectives on technology development, holding different titles and originating from distinct professional networks separate from the networks held by the authors.

The group whom we refer to as ‘Navigators’ represented captains and chief mates working aboard two ferries outfitted with state-of-the-art automation technology. All navigators had a ‘D1’ deck officer license, the highest maritime navigation license in Norway. At the time of writing, the number of navigators working aboard partially automated ferries represented a small population. As such, we were careful to characterise them broadly to avoid de-anonymising them. The *Korsvika* (a pseudonym) was, at the time of this writing, the world’s only ferry operating regularly with both auto-crossing and auto-docking, making it relatively easy to identify. It was on this ferry that field observations took place. There is a total of eight deck officers on the *Korsvika* and we interviewed three of them. The second ferry in our case study, called the *Vikhammer* (also a pseudonym), had just auto-crossing installed. The *Korsvika* and *Vikhammer* were owned and operated by different companies.

### Data analysis

Our analytical approach was inspired by Grounded Theory Methods (Corbin and Strauss, 2015; Glaser and Strauss, 1999; Morse et al., 2009). Observations made on the navigation deck aboard the *Korsvika* also served an important role in the analysis, describing the context in which navigation work took place. During interviews and field observations, insights were recorded as ‘memos:’ dated text excerpts ranging from short notes to long, descriptive passages. No less than 101 memos were recorded in total, which served as precedents to a more structured analysis aimed at synthesizing these early insights.

In structuring the analysis, we used the software tool NVivo (NVivo, 2020). At its most fundamental level, the analysis comprised of ‘codes’ – units of highlighted text representing potentially relevant findings. Our analysis consisted of several hundred codes, which we assigned to categories called ‘axial codes.’ Special attention was afforded to retaining terms and phrases used by informants and to resisting re-interpretation in our own wording. For example, the term ‘backup,’ emerged as an important axial code. While only two navigators used the term expressly, the saliency of the theme was made apparent through other related codes (e.g., Ola: ‘you become an operator who monitors the systems and is ready to press a button if there’s a bug;’ Henrik: ‘When what you see on the screen no longer shows the correct thing, that's when things get interesting’). The axial coding process was iterative and was conducted by the first author and two graduate students, involving many rounds of discussion with the authors over the study period.

Eventually, we distilled our analysis into an overarching narrative structure, focused on the discrepancy between designers’ construal of navigators’ work and navigators’ own reflective accounts. These findings are presented in Sect. 5. Before presenting this, however, it is necessary to provide some context to the findings. In Sect. 4 that follows, we outline the work activities making up a regular crossing aboard the *Korsvika*, constructed from first-hand field observations.

### Methodological limitations

The empirical study consisted of both field observations and semi-structured interviews, lending our research design some distinct advantages as well as limitations. One advantage, for instance, presented itself from conducting independent, one-on-one interviews, as it led to the discovery of discrepancies between designers’ and navigators’ accounts of the same core activities. Similarly, this approach lent itself to making comparisons within groups. For example, when we compared accounts of navigators on different ferries, insights emerged linking their use of automation with skill degradation (Sect. 5.1). The conditions of confidentiality and anonymity, too, proved to be helpful in a way that field observation alone could not be. Informants were free to express their opinions without the potentially self-censoring effect of their colleagues’ or managers’ presence and reflected on their work activities as if observing them from the outside. Having interviewed the operators during a global COVID-19 pandemic, video conferencing provided a useful platform for data collection during social distancing.

## Field observations aboard the *Korsvika*

**Figure 2:**
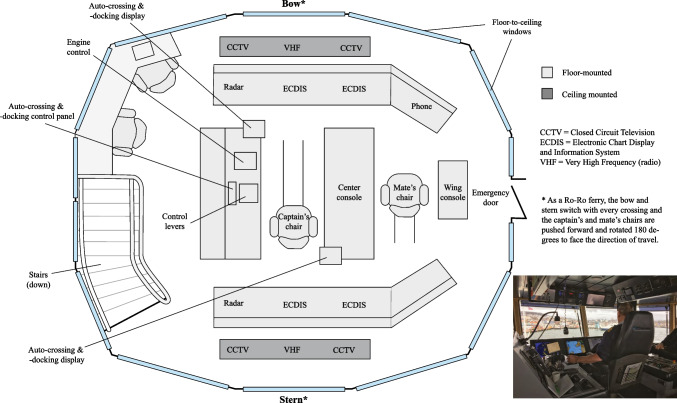
Layout of the bridge aboard the *Korsvika* (image taken by first author)

In this section, we present field observations from the *Korsvika*. The *Korsvika* (a pseudonym) is the world’s first ferry in regular service equipped with auto-crossing and auto-docking: two technologies representing a step change in the transition towards higher levels of machine autonomy in the maritime domain. For simplicity, we refer to the two technologies together as ‘auto-systems.’ The account that follows is a description of work during a regular crossing, as well as the environment of the navigation deck and the functionality of the auto-systems. The aim is to provide context about the roles, responsibilities, and tasks of the navigators, how these navigators interact with each other and the auto-systems, and how the adoption of higher levels of autonomy impacts their work activities. The diagram in Figure 2 can be used to orient the reader on the *Korsvika*’s navigation deck.

### The *Korsvika*’s ferry service

The *Korsvika* connects vehicle traffic and foot passengers between two busy ports in Norway. The crossing takes less than 45 minutes. Operations are going smoothly when this 140-m-long roll-on/roll-off ferry, with capacity for almost 600 passengers and 200 cars, is on time with an even gap behind the other ferries that sail the same route. Because several ferries traverse the same crossing, issues can arise when one ferry is delayed, forcing the ferry behind to wait for it outside the dock. There are many factors that can affect the ferry’s service, including the weather conditions and even the sailing styles of different navigators on duty. The new auto-systems installed aboard the *Korsvika* were intended to improve the efficiency of ferry service, saving fuel while providing customers with a more consistent service.

### The *Korsvika*’s crew

The captain has overall responsibility for the safety of passengers and crew. The chief mate (often shortened to ‘mate’) shares much of this responsibility. The captain and mate relieve one another’s shifts throughout the ten-and-a-half hour working day, exchanging regular handovers in what the navigators call ‘sharing a voyage.’ Two bosuns handle the physical work on the main deck: loading, unloading, fitting cars, maintenance, and checks of safety equipment. One of the two navigators (captain or mate) communicates to the bosuns over a local radio and observes their actions from the bridge windows or on Closed Circuit Television (CCTV). The shipowner requires that two crew personnel must always be on the bridge, so after handovers between the captain and mate, a bosun comes up to the bridge and joins as a lookout. Other than the navigators and bosuns, the crew consists of a chief engineer, a mechanic, and cafeteria crew. Of all the crew aboard the *Korsvika*, the new auto-systems directly affect only the navigators’ day-to-day work.

### Loading and leaving dock

At the dock, the ferry loads vehicles and foot passengers. When loading is completed, the command ‘Lift up!’ is radioed to the bosuns, cueing them to close the ramp door and secure it for crossing.

Leaving the dock can be accomplished by the navigators either manually using thruster controls (‘at the handles,’ to use their terminology) or by pressing a button on the new auto-docking system. Currently, the auto-docking is used for 50–70% of all voyages.

Leaving the dock, the captain or mate reports their departure to the local Vessel Traffic Services (VTS) centre that they have left the dock, and VTS replies with any relevant information about traffic in the area. The navigator also keeps an eye out for small recreational boats, which are typically not detected by VTS. The new auto-systems are not yet equipped with cameras to detect possible collision targets, so the navigator must be attentive even when in auto-mode.

### A regular crossing in ‘auto-mode’

Shortly after the ferry is clear of the dock, auto-crossing is engaged by pressing the ‘AUTO CROSS’ button on the console. Nearby, on a small screen the size of a tablet computer, a touchscreen indicates that auto-crossing has been engaged and displays system information like thrust and heading. The handles on the thruster controllers move by themselves as the ship settles into its route and adjusts its speed for the crossing. The captain sits back in the chair and looks out the window, occasionally glancing at the Electronic Chart Display and Information System (ECDIS) and radar. The lookout sits in the mate’s chair beside the captain, looking out the window and glancing occasionally at the captain.

Sometimes, small boats are encountered enroute. On weekends and summer holidays, there may be many such recreational boats in addition to regular commercial traffic. These small boats warrant special attention, because unlike commercial ships with trained crew, their occupants may be unfamiliar with the rules of navigation and may occasionally end up on a collision course. The auto-crossing is not yet capable of avoiding collisions. Avoiding collisions remains one of the core duties of the navigators. For larger ship traffic, whose navigators manoeuvre their vessels in accordance with Collision Regulation conventions (COLREGs), there are generally no issues avoiding collisions. Should a ship cross from either port or starboard, an agreement is usually made over the radio regarding who will adjust course or speed to pass behind the other, even if it is the give-way vessel that does so. In a give-way situation, the navigator takes over manual control by pressing the ‘MANUAL’ button on the auto-system console. Pulling back on the thruster, the other ship can cross ahead, whereafter the navigator can press ‘AUTO CROSS,’ resuming the crossing and losing little time to the timetable.

### Arriving at dock and unloading

Approaching the dock, the auto-system alerts the crew with a loud beep followed by a pre-recorded voice announcing that docking is about to start. The alarm is acknowledged by the captain by pressing the ‘AUTO DOCK’ button that starts the auto-docking stage. Were the captain to ignore the alert, a safety measure is built in to stop the ferry in station-keeping mode, holding position some distance away from the dock.

As the ferry heads to the dock slowly under auto-docking control, the mate joins the captain (or vice-versa) in time for the docking sequence. At this point, the bosun who was on lookout duty during the crossing heads down to prepare for unloading. ‘Betty’s taking care of it,’ announces the captain, using a nickname referring to the auto-docking system. The mate acknowledges, confirming they understood that the ferry is docking automatically.

At the dock, the captain communicates with the bosuns over radio and the ramp is lowered and unloading commences. Shortly after unloading, loading begins again. The captain’s and mate’s chairs are slid forward and rotated 180 degrees and the *Korsvika* sets out for its other port in the direction from where it came.

### Higher levels of autonomy and centres of coordination

Currently, there are cameras installed in the *Korsvika* bow that record all marine traffic it encounters. Technology developers behind the auto-crossing and auto-docking initiatives are working towards enhancements; for example, they can use the recordings to train machine learning algorithms that can classify objects and be used in collision avoidance algorithms. As development of more advanced automation continues, there have been discussions about reducing crew aboard the ferries and controlling fleets of highly automated ferries from a land-based centre of coordination. Higher levels of autonomy have already proven successful on the *Korsvika*, improving the efficiency of fuel consumption and consistency of service in the face of highly variable external factors. Unlocking the potential benefits of higher levels of machine autonomy, though, depends on seamless integration of the AI systems with what is, at its heart, human work.

## Interviews with designers and navigators

In this section, we present the findings of the interviews with navigators both aboard the *Korsvika* and the *Vikhammer*, as well as with technology designers and researchers shaping the transition towards higher levels of autonomy in the maritime domain. We start with the navigators, who recounted a shift to a ‘backup’ role subsequent to the introduction of auto-systems aboard their ships. Then, we compare this to accounts of the designers, whose construal of working with automated systems seemed misaligned with navigators’ own accounts of working with automation in the field.

### Navigators’ perspectives: shifting to a backup role

The navigators attributed agency – a capacity for action – to the auto-systems. The influence of this agency was most evident in their descriptions of transitioning from ‘hands-on’ to ‘backup’ navigation.

The nickname assigned to the auto-systems by some of the navigators (‘Betty’)exemplified how machine agency could be manifested. Betty could ‘take care of it,’ as Robin reported, referring to the complex process of docking the 1400-ton *Korsvika* to the dock. In fog, Betty was ‘ingenious’ given her ability to dock in zero visibility. Betty could be a ‘nag,’ however, and ‘do weird things,’ according to Ola, who, as if by way of assuring themselves, told us that ‘she has no thoughts of her own.’

Robin: ‘My captain and I, if we’re auto-docking, we say that “Betty’s taking care of it.” Then he knows that auto mode is on. If we have normal autopilot on then I say that “Betty’s not taking care of it.”’

The nickname ‘Betty’ was used by two of the five navigators we interviewed, both aboard the *Korsvika*. Traditionally a woman’s name, Betty was chosen owing to the system’s female voice announcements, played at intervals to announce stages of operations or to alert navigators’ attention to some procedure or sequence. Personified in this way, the navigators described interactions with the auto-system in human terms.

The agency attributed to the auto-systems underpinned the emerging ‘backup’ role described by the navigators. We adopted the term ‘backup’ from Robin, who, describing a transition in their work in recent years, said, ‘We are the backup if something happens.’ Other navigators described a similar role. ‘You go from being the one who performs something to just monitoring something,’ said Henrik of the transition, ‘but when what you see on the screen no longer shows the correct thing, that's when things get interesting.’

One limitation of the auto-crossing was that it did not yet have automated collision avoidance capabilities, meaning such manoeuvres were left to navigators. Collision avoidance manoeuvres are regulated in the 1972 Convention on the International Regulations Preventing Collisions at Sea (COLREGs). The convention lays out traffic rules, like Rule 8 stating that collision avoidance actions must be ‘made in ample time and with due regard to the observance of good seamanship’ (IMO, 1972). Rules work best if everyone knows them, which is not always the case. ‘The biggest problem is with small boats and sailboats,’ Ola reported. ‘They don’t have the same knowledge about rules, speed, and direction,’ explained Henrik. ‘They think we move slower,’ said Robin, ‘so we have to press “MANUAL” … you don’t want to run someone over.’ In collision avoidance, the navigators’ backup role to the auto-systems was clearly defined: take over control to adhere to the COLREGs, with special attention to small boats. Another backup role emerged, however, with less clearly defined parameters. This was illustrated by Robin who recounted an instance when they took over control to make a crossing more comfortable for passengers:

Robin: ‘… these days we [the navigators] say: if it’s blowing, we steer manually. Auto-crossing can be used at any time, but manual mode is more comfortable for passengers.’

Interviewer (Erik): ‘You steer the ferry [manually] so it's more comfortable for passengers?’

Robin: ‘If you have rolling, people can fall and hurt themselves. Instead of rolling all the way over, I sail a little North and then a little South to go across the waves.’

Robin’s interaction with the automated system in this case is not determined by safety–critical and timely intervention, but rather on the system’s inability to account for comfort of passengers. Whether in taking over control to avoid hazardous traffic situations or simply to attend to passenger comfort, the shift from a hands-on role to a backup role underscored the most salient change in navigator work after the auto-system’s introduction.

One effect of shifting to a backup role was skill degradation associated with more time spent in a passive, monitoring role relative to hands-on, manual control. Skill degradation was especially apparent when comparing navigators’ accounts from the *Korsvika*, who reported that 50–70% of crossings were in auto-mode, to the *Vikhammer*, who reported close to 100% automated crossings. As reported by Henrik, the crew of the *Korsvika* had taken to driving the ship manually ‘at least twice per shift so as not to forget how that works.’ This suggested that skill degradation set in quickly, possibly over the course of days, and that regular practice was an effective countermeasure. ‘…when I have driven a lot of auto,’ said Henrik, ‘I have to steer a couple of times myself to get the feel of it again.’ Robin expanded on the subject, noting that operators’ propensity for regular manual sailing practice resulting in it being incorporated into the shipowner’s operating procedures:

Robin: ‘We’ve set it up so you’ll sail it [the ferry] yourself during the day to maintain your driving. That’s written in our procedures now. If you’ve had a holiday, you’re allowed to steer the whole shift, there’s no one that says you have to use auto-crossing.’

On the *Vikhammer*, in contrast, the crew had seldom sailed manually since the auto-crossing was implemented. This implied a more significant skill degradation, which might compromise safety in the eventuality of a manual takeover.

Magnus: ‘We only use auto-crossing now – every day, every trip.’

Interviewer (Erik): ‘Do you ever turn it off to take manual control?’

Magnus: ‘No.’

Interviewer (Erik): ‘When was the last time you drove manually?’

Magnus: ‘We might occasionally drive if we have an ambulance dispatched. Auto-crossing must have the lowest energy consumption, but with an ambulance it’s life and health. Apart from that … it’s been one-and-a-half years since I stopped doing it [driving manually] myself.’

Given how fast de-skilling was a factor among the crew of *Korsvika*, one cannot help but wonder if the crew of *Vikhammer* are prepared for an ambulance dispatch. Manual skill practice procedures, even in situations well-suited to the automation, appeared to be a useful countermeasure to skill degradation for the navigators aboard the *Korsvika*.

### Designers’ perspectives: prescribing action for distributed work

The interviews we held with technology designers and researchers yielded insights into how development activities are shaping the transition to increased human-AI collaboration in maritime navigation. Here we outline what this group identified as the most important design goals and what methods they are adopting to address interaction challenges between humans and machines. Then, we compare designers’ construals of working with higher levels of machine autonomy with navigators’ own corresponding accounts.

To begin, we outline some of the major design goals, the approaches being adopted in the industry and research communities, and what specific challenges represented outstanding gaps and issues. The main goals driving the transition towards higher levels of autonomy in the maritime domain included achieving improved ‘logistics,’ ‘system design,’ and ‘centralized control.’ These goals, it was envisioned, will be accomplished primarily through crew reduction relative to ship payload, as well as through centralized management of employees and ship assets from a centre of coordination. ‘The whole problem statement,’ said Vidar, ‘can be defined as moving work farther from the pointy end to more distributed locations.’ By ‘pointy end,’ Vidar referred to operational work in the field, a term coined by organizational scientist Rhonda Flin (Flin et al., 2008) and used often in the context of exposure to hazardous working environments. Asked to describe the vision of autonomous ships, interviewees described fleets of ships with reduced crew (or in some cases, no crew at all), whose whereabouts were tracked by trained operators in a centralized control centre. Prompted further to describe the control room, images of data-rich information displays were invoked in all interviews (‘there will be large-screen displays displaying the “big picture,”’ reported Karl; ‘through the screen [the operators] will have access to the data they need,’ said Andreas). Many of the technological artefacts located in a conventional ship were mentioned, including ECDIS, marine radio, and software for ship scheduling and voyage plans. What distinguished the control room from ship’s bridge was the amount of additional data (e.g., video streams, sensor displays, and the like) and, crucially, the ability to take direct control over the ship. Here the analogy was made by five of the interviewees to VTS operators, who, tasked with monitoring traffic in busy port areas, can indirectly direct traffic by contacting navigation crew over radio. In a control room for highly automated ships, such actions could be taken directly instead of indirectly, making the control room operator effectively a remote captain in addition to traffic director.

Two interviewees described interactions at the screen interfaces in terms of ‘top down’ and ‘bottom up’ processing. As explained by Karl, this was intended to support decision making at the cognitive level, combining top-down processing (‘information search’) with bottom-up driven processes (‘information that catches the attention of operators’). Two opposing viewpoints emerged, however: some interviewees argued that the control room should be designed to accommodate work as it takes place aboard a ship’s bridge; others argued that the control room will require a ground-up approach, requiring specifications drawn up according to distinct requirements. Among the latter group, ‘human-centred design,’ ‘prototyping,’ and ‘systems engineering’ featured as methodologies to uncover these distinct requirements. Discussions about design strategies met a significant challenge: for highly automated ships, there were no standardised guidelines aimed for accommodating approval like those akin to conventional ships. Conventional passenger ship design, for example, is standardized according to design guidelines laid out by classification societies like DNV GL in their ‘Rules for Classification’ (DNV GL, 2017). For highly automated ships, adopting ‘goal-based approaches’ were, in place of prescriptive approaches, the most viable option towards approval of designs by regulating authorities. Characterizing this goal-based design process, five interviewees called it a ‘transition,’ involving testing, verification, and approval – lengthy processes typical in the highly regulated industry of shipping.

The technology for enabling high levels of machine autonomy, it appeared, was more or less available; orchestrating this technology in a real-world context, though, remained the challenge. In the boundary between human and machine, several gaps and issues were identified. The number of vessels, for instance, that each operator should control was unknown. This number was linked to the LoA of the vessel, but the LoA, too, was ambiguous, referencing various taxonomies each with its own configuration of how automated tasks and human tasks combine to navigate a ship. Specific LoA taxonomies mentioned by the interviewees included DNV GL (2018), IMO (2018), and NFAS (Rødseth, 2017). A central problem was the amount of time it takes to take over control. On the premise that such control takeovers are preventative and time-critical, the maximum allowable takeover time emerged as perhaps the single most important factor in goal-based design directed towards collaboration with the automated system.

Tommy: ‘You must quantify the person’s response time. This will help a lot with the approval of a shore control centre, because then you can document, for example, that the system gives ten seconds warning and that we have done the research showing that the operators are trained for this. Today, nobody knows.’

### Discrepancies between navigator and designer accounts

Comparing interviews of designers and researchers with those of navigators, certain discrepancies came to light. Two such discrepancies pointed to ways in which designers’ construal of human–machine interaction diverged from those who inhabited this interface in their work. The first related to how the two groups treated decision making for control takeovers; the second related to how they reconciled their safety responsibilities while relinquishing tasks and decision making to machines.

Designers, in their efforts to build interfaces that supported navigators’ work, adopted practical models for decision making based on sensory input and cognitive processing. The model of ‘situation awareness,’ attributed to Endsley (1995), was especially prominent, appearing independently in four of the nine interviews we held with designers. Navigators, by contrast, did not refer to situation awareness, neither directly nor by its characteristic features, which decompose decision making into distinct information processing stages. In the following excerpt, for instance, Karl, a designer, described design needs for a control room to support work for navigating highly automated ships, framed in terms of ‘situation awareness’ needs:

‘What data is needed to control and monitor the [highly automated] ships: that is situation awareness need number one. Then situation awareness need number two is to display that into something understandable. Situation awareness need number three is to project that into the future. That could be a way to approach the concept [of operating highly automated ships] in a more… systematic way, perhaps.’

By contrast, navigators invoked heuristic approaches to decision making, drawing from in-situ skills informed by experience. One example of such a heuristic was illustrated by Robin who recounted taking over control to attend to passenger comfort (see Sect. 5.1). In that example, rather than following a sequence of information processing stages, the decision to take over manual control stemmed simply from imagining how passengers would experience the crossing in the given sea state.

Four of the nine designers we interviewed expressed the concept of being ‘in the loop,’ referring to the state of mind one must be in to take over control from automation. The navigators, by contrast, referred to this same state as ‘backup.’ Being backup reserved the sense of responsibility that comes with being a navigator, while losing the agency involved in manoeuvring a ship under one’s own hand (‘The job hasn’t changed,’ reported Ola, ‘but in auto you can sit back and let the system do it’). Being ‘in the loop,’ by contrast, construed the navigators as components in a larger, cognitive system. In this ‘loop,’ whose terminology is rooted in control theory, the navigator was expected to passively monitor the closed loop of automated control and immediately close this loop – through timely and decisive takeover action – the moment the loop’s integrity was compromised. As explained by Alexander, a designer, ‘The key challenge will be to get the operator, in the shortest possible time, to get in the loop of what is going on.’

## Discussion

In this section, we explore the implications of the field observations and interview study results, framed in terms of the knowledge gaps and issues introduced in Sect. 1 and outlined in more detail in Sect. 2. Towards this aim, we focus discussions around three relevant themes: (i) the agencies of humans and machines in collaborative navigation, (ii) the transition to centres of coordination, (iii) the social implications of AI collaboration, and (iv) control rooms of the past, present, and future.

### Agencies of humans and machines in collaborative navigation

One of the most salient themes uncovered in the analysis was a transition to a ‘backup’ role, defined by peremptory control interventions, or ‘takeovers.’ For technology designers and researchers, the transition toward higher levels of autonomy in shipping culminated in centres of coordination, where operators were ‘in the loop’ of the system. Navigators’ accounts of inhabiting this transition in their own work reflected a preoccupation with their own agency and expressed a desire to recover this agency. Backup implied two mutually exclusive activities: passive monitoring in situations for which the automation was well-suited, and active control in situations for which it was not. Backup invoked the ‘sorcerer’s apprentice’ role (Sect. 1), necessitating timely intervention to stop the conjured force of a machine imbued with agency.

Lucy Suchman, in her ‘Plans and Situated Actions’ (Suchman, 2007), framed the human–machine interface in terms of co-existing intentions entrenched both in control algorithms (plans) and in-situ skills (situated actions). In the context of the backup role, navigators co-existed as passive operators when plans represented by the automation proceeded as expected, and as skilled operators when those plans were inevitably jettisoned to deal with some situation at hand.

Navigators’ accounts also underscored the extent to which the canonical ‘ironies of automation’ applied to the present transition (Sect. 2.4). One such example emerged from the observation of skill degradation in navigators’ ship-handling (Sect. 5): the auto-systems were, in effect, compromising the very thing it was designed to improve. Given the central importance of in-situ takeovers in the backup role, the manual ship handling skills seemed, paradoxically, of heightened importance in the face of increasing levels of machine autonomy.

As part of the backup role aboard the *Korsvika* and *Vikhammer*, there was a sense that in order for operations to go smoothly, navigators depended on the automation system as much as the automation system depended on the navigators. While the auto-crossing and auto-docking systems onboard represented relatively low levels of autonomy, the stakes introduced by this inter-dependence appeared to be getting higher for higher levels of machine autonomy. Demski and Garrabrant (2019), envisioning the system requirements for an ideal cooperative AI, called this inter-dependence ‘embedded agency.’ By this design, a cooperative AI must be self-referential, capable of modelling is own impact on its environment, including how its users adapt to its presence. Dautenhahn (2007) framed this same capacity in terms of social interactivity, pointing out that activities requiring increasing degrees of interactivity require the computational system to be able to reflexively adapt to constantly changing conditions – a form of artificial ‘social’ intelligence. Navigation is exemplary of such an activity, requiring attention not just to what tasks can be automated, but how they should be automated in the context of a socially organized activity.

Whether it was framed as ‘in the loop’ by designers or ‘backup’ by navigators, being continually prepared for takeovers emerged as the defining feature in a new landscape of joint human-AI agency. The takeover, which symbolized the boundary between machine and human control, helped bring to light two specific design issues: firstly, operators’ sense of agency was upended, manifesting in skill degradation over longer periods of passive monitoring; secondly, effective collaboration between operators and highly automated navigation systems was left hanging in the balance of situated actions and computational plans in a flux of changing situations.

### Transition to centres of coordination

The need for supervisory control of highly automated ships has generated renewed interest in centres of coordination for marine navigation. Referred to as ‘shore control centres,’ ‘remote operating centres,’ or ‘remote control centres’ by the informants in our study, centres of coordination of this type have emerged only in the past decade and have since grown significantly in the scientific literature (Veitch and Alsos, 2022). This renewed interest warrants a closer look at the guiding principles presented in Suchman’s original articulation of the centres of coordination concept (Suchman, 1997), which was aimed especially for designers (see Sect. 2.2). In revisiting the theoretical considerations associated with centres of coordination, we also ask whether they are still relevant given the recent technological developments in the decades following the concept’s introduction.

To begin, it is worth reiterating how centres of coordination relate to maritime navigation and to the transition to higher levels of machine autonomy. After all, the original case used to characterise them encompassed airline ground operations, a domain distinct from shipping both in sociotechnical and cultural aspects. Regardless of the differences, however, many of the core elements of centres of coordination were reified in the ‘shore control centre’ case. Specifically, the need to orient workers to the emerging requirements of safety- and time-critical situations was front and centre. The emphasis of locating technology use within socially organized activities, too, was of central interest, as were the requirements for workers to maintain competencies in reacting appropriately to emerging situations. Additionally, like in airline operations, the marine operations had to be orchestrated across different locations (e.g., port authorities, Vessel Traffic Services, other ferries and ships) and in line with a timetable.

The treatment of technology interactions as a strictly material practice, however, should be re-evaluated in the context of highly automated ships. Centres of coordination originally laid out technologies as an ‘assembly of heterogeneous devices’ (ibid., p. 44), placing the locus of particular actions at particular technological artifacts. Observing technology trends towards openness and interconnectedness, Monteiro et al. (2013) shifted this locus from mere ‘artefacts’ to ‘information infrastructures,’ showing the latter were distinct by virtue of networks that obscure any fixed notions of user, and even time or place of use. Recently, scholars have shifted the locus of interactivity even further from the material boundary, attributing not just agency to computational systems, but also the capacity to enact this agency in their natural environment – conditions allowing for the emergence of behavioural characteristics (Rahwan et al., 2019). Researchers in the field of ‘machine behaviour’ correspondingly describe as their mission ‘the study of ways in which introduction of intelligent machines into social systems can alter human beliefs and behaviours’ (ibid., p. 483). Experiments using games have already indicated that interacting with algorithms can increase human collaboration and may even improve group performance (Crandall et al., 2018; Shirado and Christakis, 2017). Whether the same holds true for work collaboration and navigation activities, though, remains uncertain. 

Despite this shift away from the materiality of technology interaction, the core issues associated with centres of coordination raised several decades ago by-and-large still apply for today’s transition in maritime navigation. Whether framed as artefacts, information infrastructures, or enacted AI agents in the CSCW sense, the interactivity of technologies in socially organized activities is still met with an inherent ‘otherness’ from their human collaborators. Moreover, the degree of interactivity is accentuated, rather than attenuated, for higher levels of machine autonomy.

### The social implications of AI collaboration

The discrepancies we observed between designers and navigators at the human–machine interface (Sect. 5.3) reinforced the need to reorient design activities towards improved incorporation of user feedback and in-situ observation. Here, we briefly examine the role of social dynamics in this design reorientation, discussing the extent to which discrepancies can be addressed by a better understanding of social implications of AI collaboration.

Discrepancies that arose in designers’ and navigators’ interview accounts betrayed the ostensible straightforwardness of how decisions are reached in day-to-day work. Work, for navigators, did not unfold as a neatly distilled, stagewise process, as inferred by designers. Rather, the navigators invoked a more intuitive, heuristic decision making based on common sense and tacit knowledge gained from experience. Reflecting on their role, the navigators were more than just ‘in the loop’ and ready to take preventative action. They were custodians of the automated system, presiding over its operation and arbitrating in its decision making capacity in the context of real-world events. The question of how to address this gap, though, remained largely open.

Methods employed in CSCW may provide useful tools for addressing design challenges presented by developing more aligned collaborative systems. These methods, in contrast to the more prevalent cognitivist and computer science perspectives in AI systems design, consider the sociality of technology use in its natural setting. As Bødker (1991) observed with engineers immersed in computer-aided drawing, the interface between human and computer can become a site in its own right, with its own physical form and possibilities. Revisiting the seminal CSCW control room studies of the 1990s and early 2000s sheds light on how this type of site can form within a socially organised, collaborative setting (see Sect. 2.2). Extending these studies to the case of AI collaboration, what Heath and Luff (1992) described as ‘mutual monitoring’ in line control rooms, for instance, may be recast in the present context. Mutual monitoring originally involved instances where operators divided their attention between their own tasks and the perception of colleagues’ actions through myriad cues, signals, and gestures – subtle yet essential coordinating actions in their work. A parallel can be drawn to modern ‘explainable AI’ (XAI) techniques, where one strategy involves generating heatmaps tracing where image recognition is ‘looking’ when classifying an image. In one such example, a machine learning algorithm trained to assist physicians diagnose skin cancer was designed to output details about what pixels it was analysing to reach its predictions (Esteva et al., 2017). Output in so-called ‘saliency maps,’ the algorithm in effect showed its collaborators ‘where it was looking.’ A recent review suggests that such collaborative approaches in diagnostics leads to better performance than either physician or AI working alone (Tschandl et al., 2020). Efforts like this are in line with the ‘cooperative eye hypothesis,’ a theory positing that humans evolved to have large sclera (whites of the eyes) to enable them to follow the gaze of others in cooperative activities, favouring selection of those able to coordinate communicative interactions (Kobayashi & Kohshima, 1997, 2001; Tomasello et al., 2007). Following this logic, enhancing explainability by ‘showing where the AI is looking’ may be considered among XAI efforts shifting to a more social view of computer interactivity, efforts whose merits are also recognizable in the collaborative control room setting from previous generations of ethnographic CSCW studies.

### Control rooms of the past, present and future

Examining control rooms of the past and present (through literature review, expert interviews, and comparisons to other domains) has compelled us to make inferences about control rooms of the future. Here, we briefly discuss the extent to which such future explorations are rigorous and valid in the sense typically invoked by scientific research. We make the case that despite the speculative nature of our results, they constitute relevant contributions to the CSCW and design communities through their ability to articulate an under-constrained problem and generate design insights.

Our study results were speculative because, although grounded in expert interviews and field observations, they were exploratory in nature and aimed to generate rather than converge new design ideas. The starting point for the research was not a clearly defined problem calling for a clearly defined procedure; on the contrary, it was an under-specified problem calling for a correspondingly open-ended approach. In the design community, such problems often call for a ‘research through design’ approach (Frayling, 1993), where the goal is generating ideas through a range of pragmatic and conceptual insights. As intimated by Frayling, design is concerned with ‘the new,’ and as such has a close relationship with research despite the futility of its meeting the rigorous standards of a scientific research method. Inspired by Frayling’s thinking, Zimmerman et al. (2007) defended ‘research through design’ approaches based on their propensity to produce the ‘right thing’ in the face of under-constrained problems. The approach’s underlying contribution, they argued, was based on the strength of its potential to ‘transform the world from its current state to a preferred state.’ It is partly this preferred state that is so important for the researcher to articulate. In this article, we described the preferred state of future control rooms for highly automated ships through expert interviews as well as through literature review and comparisons to other domains. Set into a multi-disciplinary conceptional framework, this articulation is among the main contributions of the work, asserting that in order to effectively address a problem, it is necessary first to formulate the situation at hand. In this case, it was especially elements of social interactivity in future control rooms that was articulated (who will work in the control rooms, and what will it be like to work with increasingly automated systems?).

Similar scholarly approaches have been applied to design of centres of coordination for highly automated ships, where ‘future workshops’ stand out as a popular approach (e.g., Hoem et al., 2022; Lützhöft et al., 2019). In this approach, experts are invited to discuss open-ended issues under the pretext of informing design activities. What future workshops have in common with the expert interviews we utilized is that they both imagined future sociotechnical systems that fit a defined situation, shifting the focus from generating tangible solutions to eliciting insights and generating a better understanding (Lindley and Coulton, 2015). Such methods necessarily yield ambiguities, which, like Frayling’s ‘research through design,’ reflect results that, as expressed by Gaver (2012), are ‘provisional, contingent, and aspirational.’ Yet, the strength of such results lies in its exploration of real issues, gaps, and opportunities, as well as in its ability to articulate the situation at hand. In this sense, the rigour and validity of such future explorations lie in its relevance, however speculative, to designers shaping that future.

### Conceptual limitations and future work

Our practice-based research consisted of interviews in addition to field observations more in line with the CSCW tradition. While ethnographic methods remain indispensable in CSCW research, the addition of interview-based methodologies in our case helped to open the self-contained nature of what Monteiro et al. (2013) call the ‘here and now’ of field studies. Acknowledging that human–machine interaction is always in transition, such an approach contributed towards a more open-ended treatment of themes that remain constant – the ‘otherness’ of machine agency, the sociality of technology use – and that offer stable reference points in an inherently transitory study domain.

There were, however, some key limitations of the research design. One such limitation involved the extrapolation of work activities in a maritime navigation setting to that of a control room, and the extent to which this extrapolation provided a representative case. At the time of this study, no shore control centres for coordination of highly automated ships existed in full operational scale. The choice of studying navigation aboard highly automated ferries was used as an approximation for the control centre case. A future shore control centre will, it was argued, be organised around the same core activities – just with higher levels of autonomy and at a remote location. The choice of studying professional design activities towards building shore control centres helped to ground this extrapolation. Although this extrapolation identified several relevant themes, future work must be tuned in to the ways in which human collaboration is affected in a real control centre environment.

## Conclusion

Maritime navigation work is in transition, marked by collaboration with increasingly high levels of machine autonomy. In this study, we framed this transition as an opportunity to study how designers are shaping work and how navigators are adjusting to the changes. Maritime navigation in this sense served as a representative case study for broader applications of safety–critical, distributed work in sociotechnical, computationally infused environments. Interviews with technology designers and navigators indicated a discrepancy between designers’ construals of working with higher levels of autonomy and navigators’ own reflective accounts of this work. This discrepancy was centred around the task of taking over control from the automation, a role designers called ‘in the loop’ and navigators called ‘backup.’ The discrepancy suggested a need to realign design strategies to real-world operational demands. The risks of not doing so are heightened in the face of increasing levels of autonomy and ongoing development of centres of coordination – efforts that paradoxically place more expectations on human operators, rather than less.

Considering the importance of mutual monitoring – the reflexive social articulations that coordinate work in control room environments – it was clear that collaboration with AI systems depended to a large extent on rendering computational activities more visible. Aligning with the needs of human collaborators involved displaying the AI system’s actions more transparently, akin to following the gaze of a collaborator’s eyes. Better alignment also pointed to designing AI systems that incorporate cues, gestures, and exclamations of their human collaborators. At least in theory, it may even require machine learning techniques incorporating embedded agency, reflexively adapting to adjustments of users influenced by the AI’s presence.

The main contributions of this work are positioned within the emerging field of AI alignment research. Located at the crossroads of computer science, engineering, design, HCI, and sociology, alignment research strives to understand how people can seamlessly interact with machine autonomy. CSCW, with its preoccupation with the sociality of computer use especially in work environments, is uniquely positioned to lend perspective on the transition towards centralized control centres for highly automated maritime navigation. The contribution of this work involved the articulation of the situation at hand to help align design to the preferred real-world interplays of computational plans and human actions. Methodologically, we demonstrated the combined use of literature review, expert interviews, and field observations to ground speculative design insights for future control rooms. Conceptually, the contributions raise the relevance of multi-disciplinary theoretical frameworks and reify theory from past control rooms studies and HCI considerations.

In maritime navigation as in other applications of collaborative work, improvements to efficiency and decision making are among the potential benefits of implementing higher levels of machine autonomy and AI. The extent to which these benefits rely upon seamless coordination with human supervisors, though, remains the domain of research oriented towards the implications of collaborating with intelligent machines in work’s natural settings.
